# Rotation Grids for Improved Electrical Properties of Inkjet-Printed Strain Gauges

**DOI:** 10.3390/s22166119

**Published:** 2022-08-16

**Authors:** Matthias Rehberger, Jonas Mertin, Christian Vedder, Jochen Stollenwerk, Johannes Henrich Schleifenbaum

**Affiliations:** 1 Fraunhofer Institute for Laser Technology ILT, 52074 Aachen, Germany; 2 Digital Additive Production (DAP), RWTH Aachen University, 52074 Aachen, Germany

**Keywords:** inkjet printing, strain gauges, rotation algorithm, silver ink, PEN substrate, nanoparticles, printed sensors, printed electronics, additive manufacturing

## Abstract

We report an image data driven approach for inkjet printing (IJP) to improve the electrical properties of printed metallic strain gauges (SGs). The examined SGs contain narrow conducting paths of multiple orientations and therefore suffer from two challenges: 1. The printing direction of inkjet printed conducting paths has an impact on film formation and electrical properties. 2. A loss-free rotation algorithm for IJP image data is lacking. New ways of IJP image data processing are required to compensate for quality-reducing effects. Novel grid types in terms of loss-free rotation algorithms are introduced. For this purpose, a new grid (e.g., 45° tilted) with a different grid constant is placed over a given pixel grid in such a way that all cell centers of the given pixel grid can be transferred to the rotated grid. Via straightening the tilt, the image data is rotated without interpolation and information loss. By applying these methods to measurement gratings of a full bridge with two perpendicular grating orientations, the influence on the manufacturing quality is investigated. It turns out that the electrical detuning of full bridges can be reduced by one order of magnitude compared to state-of-the-art printing by using so-called diagonal rotation grids.

## 1. Introduction

Drop on Demand inkjet printing (IJP) is a promising method for selective deposition of non-graphic functional materials. Printed electronic devices such as resistors [1,2], capacitors [1,3,4], (thin-film) transistors [5,6,7,8,9], diodes [10,11], antennas [12,13,14], photovoltaic cells [15,16,17,18], displays [19,20], batteries [21,22], and sensors [23,24,25,26,27] have gained interest for multiple applications. But despite great efforts at performance optimization regarding devices and corresponding manufacturing processes, examples of industrial utilization are still rare. An essential factor for this is the stability and reliability of the printing process. For manufacturing aspects, the up-scalability and process yield are important to overcome technical limitations and to become industrially relevant [7,8]. Previous research focused on the investigation and optimization of single droplets [28,29,30,31], lines [28,32,33,34,35,36] and areal elements [37,38,39] like rectangles. These studies addressed the adjustment of the distance between neighboring droplets, drop ejection frequency, substrate temperature and pre-treatment. In addition, adjustments in the print layout for evaporative compensation are carried out to achieve defined rectangular pattern shapes or limit morphological irregularities at intersections of touching or crossing lines [34]. Nevertheless, a variation of ink formulation was key of the investigations. For printed lines the printing direction is identified as an important parameter by Seifert et al. and a classification is introduced described as “in printing direction” (IPD) and “counter printing direction” (CPD) [33]. Sowade et al. expand the investigations and understanding by including the influence of print pattern orientations with rotation angles of a multiple of 22.5° in an IJP process [40].

Inkjet-printed metallic layers and structures exhibit a noticeable variation in properties regarding electrical conductivity. The reproducibility of achievable resistance values can vary by up to 30% [33,40,41]. For IJP of strain gauge (SG) measurement gratings, variations in electrical properties of several 10% are not acceptable. In combination with often required different grating orientations as used in SG full bridges, the influence of the printing direction is a crucial challenge.

**Figure 1:**
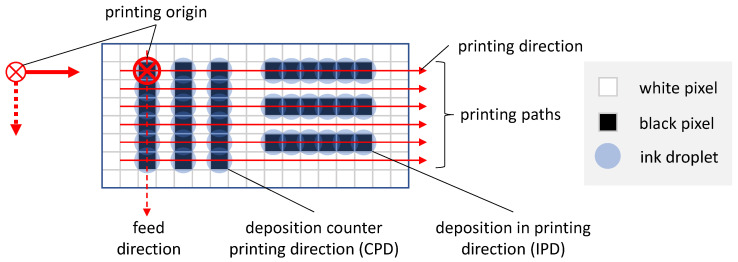
Printing origin, printing and feed direction of raster graphics in inkjet printing.

Figure 1 shows the underlying nomenclature used in this article to express the relations between printing direction, feed direction, the printing origin, IPD and CPD printing of lines. The red symbol on the left hand side in Figure 1 shows the printing origin to be in the upper left corner of the raster graphics and the printing direction from left to right. For simplification, single nozzle uni-directional printing is assumed. Thus, after finishing printing the black pixels as discrete ink droplets on the first printing path (in this example horizontal path), the printhead returns without droplet ejection. A movement is performed in feed direction (in this case vertical movement) and the second printing path (horizontal) is processed. Due to this sequence, the time difference for the deposition of neighboring droplets in printing und feed direction differs significantly. This leads to a difference in film formation of IPD and CPD printed lines as described by Soltman et al. [28].

**Figure 2:**
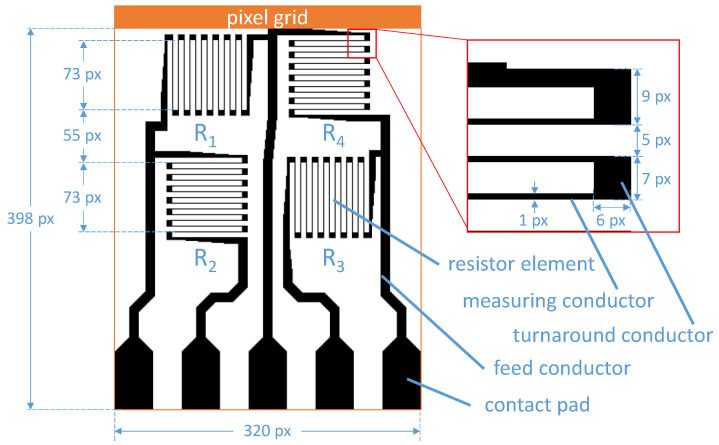
The print pattern contains a SG full bridge with two perpendicular line orientations with four resistive elements and five contact pads.

Our contribution will add a novel approach to tackle the issues arising when printing typical SG gratings containing IPD and CPD line-shaped features at the same time. This is often used in the design of SGs and is picked up in the printing pattern of these studies (cf. Figure 2). Line orientations of perpendicular alignment are commonly used in SG gratings for temperature compensation in half and full bridges. Thus, horizontal and vertical alignments of resistive segments of the grating merges the IPD and CPD printing of narrow conducting paths in a single printing pattern. To overcome the impact of the printing direction, a loss-free rotation algorithm for 1-bit IJP raster graphics unequal to multiples of 90° rotation angle is introduced. Hence, the combined IPD and CPD printing can be replaced by an “angled printing direction” (APD) and therefore reduce the effects caused by the relation of printing direction and different feature orientations.

Raster graphics are used in different ways. Depending on the type of use, different requirements are demanded of them, e.g., display on a screen, graphic printing, or printing of functional materials. The data processing development of image files is largely guided by the graphic representation of the image content. The optical impression is decisive for the human perception of quality. However, when printing functional materials, other physical properties determine the quality of the print result, such as electrical conductivity or insulation capability in the case of printed electronics. While a missing ink droplet in a printed photo is barely noticeable and hardly affects the image impression, a missing material droplet in a printed electrical conductor path can lead to an interruption of the conductive path and thus to the technical non-functionality of the entire component printed.

Typical data processing algorithms of image files are designed for the best possible representation of the optical appearance and use interpolations and a high color depth on one or more color channels for this purpose. A comparable color depth is not available when printing conductive inks, since typically only binary states—material is printed or not—are applied.

If an image consisting of black and white pixels is rotated by, e.g., 45° by means of conventional image processing for graphical representation, interpolation and the use of grayscale colors occur in order to approximate the optical appearance as closely as possible. The resolution of the image file in DPI (dots per inch) remains unchanged, while the color depth is changed from 1 bit to e.g., 8 bit, otherwise the image rotation will not be processed. The number and arrangement of filled cells on the pixel grid changes, and so does the total amount of material to be deposited and the material density distribution.

In contrast, with the lossless image rotation for IJP print files presented here, the color depth is not changed, but the resolution is. The total amount of material as well as the material density distribution remain unchanged. These are the decisive quantities and a requirement for the suitability of image transformations for IJP print files, since they take the electrical conductivity of the print material as a functional property into account.

We derive the principle of lossless image rotation for IJP using the example of image rotation by 45° and, thus, show how the algorithm works. This is followed by a formalization and a mathematical classification of the so-called u–v rotation grids for further rotation angles. In the experimental part, the algorithm is applied to an SG full bridge with perpendicular measurement grating orientations using the example of 45° rotation. The validation is performed based on the measured electrical properties in comparison to a non-rotated SG full bridge.

## 2. Materials and Methods

### 2.1. Substrate and Ink

A polyethylene naphthalate (PEN) foil (Teonex Q65HA, DuPont Teijin, Tokyo, Japan) of the size of DIN A4 (210 × 297 mm^2^) with a thickness of 125 µm and an adhesion promoting pre-treatment is used as substrate for all printing tasks. The PEN foil is covered with protection foils on both sides that are removed just before positioning on the printer substrate table.

A commercially available silver nanoparticle ink purchased from Merck (ANP Silverjet DGP 40LT-15C, Advanced Nano Products Co., Ltd, Sejong, Korea) was used for the deposition. The ANP ink is a well-known and well-established ink formulation in the field of inkjet-printed electronics and has been used by many researchers, e.g., Refs. [27,42,43,44,45,46,47,48,49,50,51]. The ink contains 30–35 wt.% of silver nanoparticles in tri-ethylene glycol monomethyl ether (TGME) as polar solvent.

### 2.2. Print Pattern

The layout of a SG full bridge with two perpendicular line orientations in the meandering mechano-resistive measurement gratings is used as a print pattern (see Figure 2). The design is laid out pixel by pixel using Adobe Photoshop CC to avoid unwanted conversion artifacts from vector graphic formats. The print file is saved as a bitmap file with 1 bit colour depth. Every measuring conductor of the individual resistor elements $R_{1}$ to $R_{4}$ has a width of exactly one pixel (px) and the 13 measuring conductors of each element cover an area of 73 × 73 px^2^. These areas are separated horizontally and vertically with an offset of 55 px respectively. Within a resistor element, the measuring conductors are connected to each other via turnaround conductors with a size of 6 × 7 px^2^. Via feed conductors the resistor elements are connected to five contact pads. The width of the feed conductors increases from the connection of the resistor elements to 10 px and finally joins the contact pads which are dimensioned with an area of 40 × 61 px^2^. The dimensions of the print file are 320 × 398 px^2^ which corresponds to dimensions of 16.26 × 20.22 mm^2^ at a resolution of 500 DPI.

The resistor elements of SG full bridges are usually interconnected in a circle and are probed via four pads. When interconnected in a circle with four contact pads, a single resistor element cannot be measured in isolation because automatically the other three elements are connected in parallel and affect the measurement result. Therefore, our design provides five pads so that each resistive element can be probed separately during electrical characterization.

The sensory function of SGs is largely determined by the measuring grating, i.e., a mostly meander-shaped electrical conductor. The shape of the grating can be divided into several segments (cf. Figure 2). Each resistor element consists of several thin, long conductive paths (line segments), which are arranged parallel to each other, so-called measuring conductors. At their ends, they are each connected by wide conductive paths (area segments), so-called turnaround conductors. This design is chosen in such a way that under mechanical deformation (compression/stretching) the change in electrical resistance of the measuring lines is as high as possible parallel to the (measuring) line orientation and as low as possible perpendicular to the line orientation. The major fraction of the resistance change is contributed by the line segments, since in the described design the relative change of conductor length and conductor cross-section is larger than that of the area elements.

### 2.3. Lossless Image Rotation or IJP of SGs of Perpendicular Grid Orientations

In additive manufacturing of meander-shaped measurement gratings for SGs with a focus on quality and reduction of manufacturing tolerances, special emphasis should be placed on the formation of the line segments. Fine structures printed by means of inkjet can be subject to a dependency on the printing direction. By fine structures, we are referring to printed thin lines (the line segments of the SG measurement grating), which are formed by stringing together individual droplets. All droplets are arranged along a line. In the raster graphics of the print file, a thin line is correspondingly a row or column of black-filled pixels, which has a width of exactly one pixel. During printing, the droplets overlap due to their diameter, being approximately twice the pixel pitch, and form a conductive path.

Moving beyond printing SG gratings of one orientation to two perpendicular orientations, an inherent challenge arises. As with the lines shown in Figure 1 on a pixel grid in two orientations, one orientation of the SG is in the direction of printing, and the other is correspondingly perpendicular to it.

The obvious solution to this effect is to print only the lines of one orientation, rotate the print direction by 90°, and then print the lines of the second orientation in addition. However, this implementation, which seems simple at first, immediately poses additional challenges. For example, the data of the print file must be processed in a content-sensitive manner and, for example, divided into two print files which are then printed one after the other. On the one hand, this involves an increase in process time compared to the conventional procedure. On the other hand, the print orientation to the component must also be changed between the first and second printing steps.

The first option is to rotate the component. Here, positioning is particularly important to precisely superimpose the print images. Positional deviations on the order of the droplet spacing (usually < 50 µm) can lead to unusable print results. The second possibility is to change the print direction via the axis system of the printer including a rotation of the printhead. If the printing system supports this mode on the hardware and software side, different linear drive axes are usually installed for xy positioning, which can differ in their dynamic behavior. If a single line is printed at a reduced speed, for example, the droplet ejection frequency must be adjusted, which in turn can affect the print result.

A change in the printing direction can thus have a negative influence on the process time and massively increase the technical requirements as well as the implementation effort (data preparation, hardware, and software of the printer). Rotation of the component or the printhead can reduce the quality of the print result if re-positioning is imprecise. Changing the print direction by rotating the printhead or substrate is therefore not considered to be effective.

#### 2.3.1. Diagonal Grids

However, the approach discussed in this paper is an alternative in which neither several print jobs are executed nor a rotation of the component or an adjustment of the printhead orientation is required. The entire structure to be printed, containing both line orientations, is rotated by 45° while retaining the set print direction. A rotation takes place in the image of the print file. In this way, two mutually orthogonal lines can be generated in the same way at 45° tilt to the print orientation in a single print job [52]. A rotation of 45° of this kind with widths of the measuring grating conductors of one pixel, can lead to such strong changes of the raster graphics that in the printed image the deposited material density on the lines varies strongly and interruptions of the electrical conductors occur. A successful application of this image transformation of line segments of only one pixel width is not known so far.

**Figure 3:**
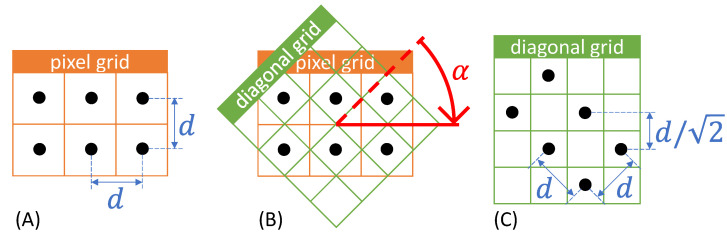
Lossless image rotation by α= 45° to a diagonal grid. (**A**) Image content on a pixel grid. (**B**) Superposition of a rotated diagonal grid. (**C**) Resulting rotated image content.

To resolve the conflict of rotation of the print to improve the electrical properties and poor mapping to a pixel grid, a new type of grid is introduced: the diagonal grid (cf. Figure 3). Rotation signifies the clockwise rotation of the image content. Rotation by an angle with a negative sign corresponds to counterclockwise rotation.

Figure 3A shows a grid with two rows and three columns on a pixel grid. The pixel grid is square, i.e., the edge length of each pixel is identical in x- and y-direction. The black dots indicate the center of each cell. In a raster graphic, the cell center represents the center of a deposited material droplet in the printed image. The distance d between the cell centers can also be referred to as the grid constant and must be set for the print image via the print resolution (in DPI). Figure 3A is a representative of an image that is to be rotated 45° without loss for IJP.

A square grid rotated by 45° can now be found so that all cell centers of the pixel grid can be represented as cell centers of the new grid. The simplest version of such a grid is shown in Figure 3B and will be referred to hereafter as a diagonal grid, since the rows and columns in the overlay shown extend along the diagonals of the pixel grid. From Figure 3B, it can be immediately understood that the positional relationships of the black dots to one another do not change and that preservation of image information in terms of the parameters relevant for inkjet printing of electrically conductive functional ink is possible. Thus, neither the total amount of material deposited nor the resulting material density distribution in the printed image change. Likewise, the changed grid constant can be derived from the superposition. Figure 3C shows the points transferred to the diagonal grid and the diagonal grid rotated by 45° compared to (B). As a result, the image from (A) has been rotated by 45° without the cell centers changing their relative spacing. Thus, the rotation is therefore lossless in the case of inkjet printing.

The reduced grid constant is taken into account in the print by increasing the resolution by a factor of $\sqrt{2}$. For a pixel grid with m rows and n columns, the number of rows and columns for the diagonal grid is respectively m+n−1. Depending on the image content, empty rows or columns may appear at the outer edges after rotation onto the diagonal grid. The image can optionally be cropped to remove empty edges.

The diagonal grids introduced here and shown in Figure 3 provide a way to perform lossless image rotation of raster graphics for IJP. While the optical appearance of a displayed image file changes, the printed image remains unchanged because the size of the deposited material droplets and their relative positioning to each other remain unchanged. This is the desired goal for lossless image rotation in IJP. Mathematically, by oversampling, any number of other grids can be found which allows the transfer of all cell centers of the pixel grid.

#### 2.3.2. 𝑢–𝑣 Rotation Grids

**Figure 4:**
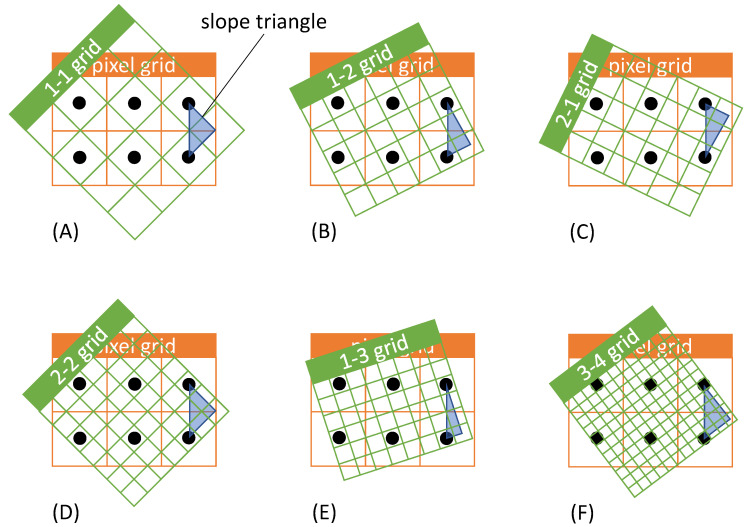
(**A**–**F**) Examples of different u–v rotation grids and their labelling.

The approach of diagonal grids and the transformation can be used to derive a general system that allows lossless image rotation through superimposed grids. For a rotation angle of α= 45°, the new grating constant is $d/\left(n\cdot \sqrt{2}\right)$ with n∈ℕ. For n=1 and n=2, corresponding superpositions are shown in Figure 4A,D. The case n=1 corresponds to the diagonal gratings described in the previous chapter. Furthermore, superimposed grids can also be found and classified for other rotation angles.

**Figure 5:**
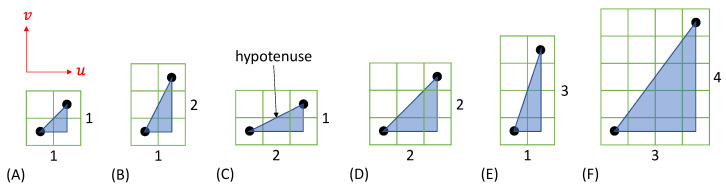
Slope triangles for classification of u–v grids for examples (**A**–**F**) according to Figure 4.

Examples of further possibilities of superimposed grids for lossless image rotation are shown in Figure 4. In the grids, a blue slope triangle is indicated in each case, which will be explained in more detail below and with the help of Figure 5. Furthermore, in the headline of the overlapping grids a denotation is given for the clear identification and classification of the grids.

Figure 5 gives the slope triangles for the six grid types from Figure 4 in the corresponding order. The black cell centers of the original pixel grid are included for illustration. The numerical values in u- and v-direction from Figure 5 indicate in each case by how many cells the points are shifted with respect to each other. These values are used for classification and labeling according to the scheme “u–v grid” (cf. Figure 4). It is given that u,v∈ℕ. From the label, the rotation angle α can be calculated directly according to

$$\alpha =\arctan\left(\frac{v}{u}\right).$$

Depending on u and v, the following cases can be distinguished for the rotation angle α:

1. u<v:  0°<α<45°
2. u=v:  α=45°
3. u>v:  45°<α<90°

Consequently, from the label of the grid, whether the rotation is less than, equal to, or greater than 45° can be read. Another important parameter is the length of the hypotenuse λ of the slope triangle, which can be calculated according to

$$\lambda =\sqrt{u^{2}+v^{2}}$$

and defines the change of the grid constant by a corresponding rotation grid. This changes by a factor $\lambda^{-1}$. This directly results in the change of the print resolution being adjusted to compensate for the changed grid constant. By changing the print resolution by a factor of λ, the original material density distribution (except for the desired image rotation) is preserved. This is referred to in the following as the resolution adjustment factor λ.

For the examples in Figure 4, the values u, v, the rotation angle α and the resolution adjustment factor λ are summarized in Table 1. The resolution as well as the resolution adjustment factor λ in this article are to be used equally for all axes of the printer.

The resolution adjustment factor λ has a crucial importance in the application of rotation grids.

- Example 1: If a diagonal grid (1-1 grid) is used for rotation (cf. Figure 4 and Figure 5, Table 1 respectively (A)), $\lambda =\sqrt{2}$. If the original print resolution before rotation is 500 DPI, the print resolution changes to $\sqrt{2}\;$× 500~707.107 DPI.
- Example 2: On the other hand, if a 3-4 grid is used for rotation (cf. Figure 4 and Figure 5, Table 1 respectively (F)), λ=5. This changes the print resolution from 500 DPI to 5 × 500 DPI = 2500 DPI.

This adjusted print resolution must be supported by the printer’s system technology. Two challenges arise: since $\lambda \geq \sqrt{2}$ holds for the described rotation grids, there is an increase in the print resolution to be used. It may not be possible to set the resolution for a particular rotation because it is too large and therefore not supported by the printer system. Another limitation may be that the printer equipment only supports dedicated discrete values for the print resolution. The PiXDRO LP50 inkjet printer used in this work correctly implements (within the measurement accuracy of the microscopy used in the analysis and the repeatability of the drive axes) the set values of the print resolution.

**Figure 6:**
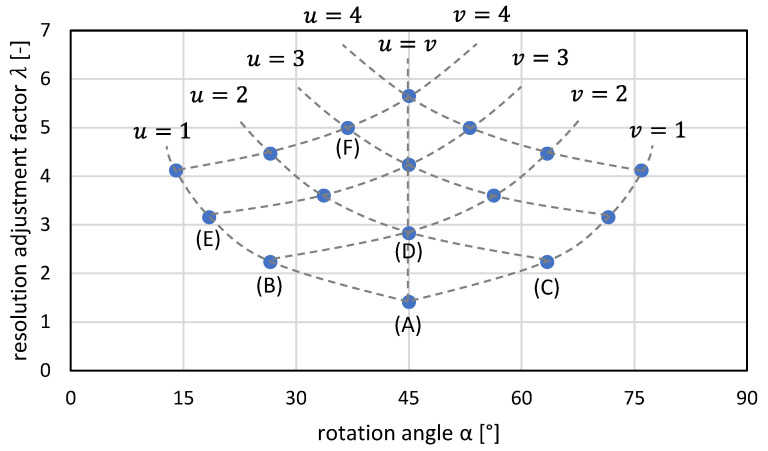
Association of the rotation angles and resolution adjustment factor for u–v grids with 1≤u, v≤4 for examples (**A**–**F**) according to Figure 4.

To get an overview of the possible solution space of the rotational grids, two parameters should be considered in detail: the rotation angle α and the resolution adjustment factor λ. Both quantities have a direct influence on the printing result and the feasibility on the printing system. In addition, they are firmly linked to each other via the u and v values. This relationship is shown in Figure 6. For all possible combinations of u and v values  ≤ 4, the resolution adjustment factor λ is plotted against the rotation angle α.

**Figure 7:**
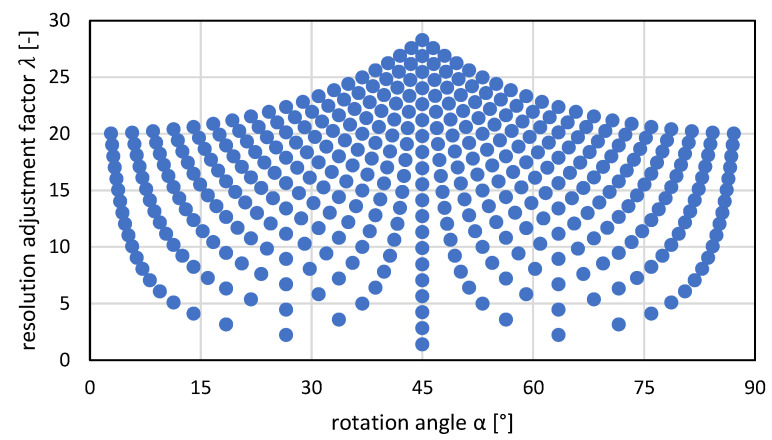
Association of the rotation angles as well as resolution adjustment factor for u–v grids with 1≤u,v≤20.

For assignment, the u and v values are plotted respectively and the points for a rotation angle of α=45° at u=v are marked on the center axis. The points are symmetrically arranged around the center axis at α=45°, which results from the underlying trigonometric arctangent function. Furthermore, the examples from Figure 4 and Figure 5 and Table 1 are marked with (A) to (F). For u and v values ≤ 20, the result is the expanded distribution shown in Figure 7.

The distribution of values with respect to the angle of rotation shows a symmetrical distribution as shown in Figure 7. However, the values between 0° and 45° and likewise between 45° and 90° are not evenly distributed. This is noticeable near the angles 0°, 45° and 90° and is due to the fractional rational argument of the arctangent function. Only by a further increase of the u- and v-values an asymptotic approximation to the mentioned angles is possible. However, this relates to a further increase of the resolution adjustment factor λ and is usually not effective for the above-mentioned reasons. Another limitation exists for common rotation angles like 15°, 30°, 60° or 75°. Their tangent is a real numerical value. Thus, it is not possible to find a fractional rational argument for the arctangent function, as required for u–v grids, which would give an exact solution for the angles mentioned. For this, only an approximate rotation grid could be found. Therefore, in this work only the simplest variant, a 1-1 grid (diagonal grid), will be dealt with in the experimental investigations.

### 2.4. Inkjet Printing and Post-Processing

IJP is performed using a Drop on Demand inkjet printer (PiXDRO LP50) from SUSS MicroTec Netherlands B.V., Eindhoven, The Netherlands (former Meyer Burger B.V.). The printer is equipped with a 128-nozzle printhead (Fujifilm Dimatix Spectra SE-128 AA) with a nominal droplet volume of 30 pl. All experiments are carried out with one nozzle, a nozzle-to-substrate distance of ca. 1 mm and uni-directional printing. The applied waveform rises the voltage applied to the piezo transducer from 0 V to 90 V in 2 µs, dwells on the maximum voltage for 8 µs and falls back to 0 V in 2 µs again. The printhead heating is activated and set to 40 °C and the ink pressure is −23 mbar. The substrate table of the printer is heated to 60 °C.

After completion of the printing process, the PEN substrate remains in the printer for approx. 30 min. until the ink has dried. Sintering takes place for 30 min. at 150 °C in a convection oven (model FDL 115, BINDER GmbH, Tuttlingen, Germany). Therefore, the coated PEN substrate is placed on a 1.5 mm thick aluminum plate for uniform heat exposure and placed in the preheated oven. After the time has elapsed, the PEN substrate is placed on a metal plate at room temperature so that the heat supply is abruptly interrupted.

### 2.5. Characterization

Optical analyses are performed using a digital microscope VHX-700F from Keyence GmbH, Neu-Isenburg, Germany. Electrical resistance analyses are performed using Keysight U1272A digital multimeter. The detuning of a Wheatstone bridge is determined from the ratio of the measured bridge voltage $U_{M}$ to the supply voltage $U_{B}$ and can be expressed by the resistances of the bridge as

$$\frac{U_{M}}{U_{B}}=\frac{R_{1}}{R_{1}+R_{2}}-\frac{R_{4}}{R_{3}+R_{4}}.$$

## 3. Results and Discussion

### 3.1. Experimental Evaluation of Diagonal Grids

**Figure 8:**
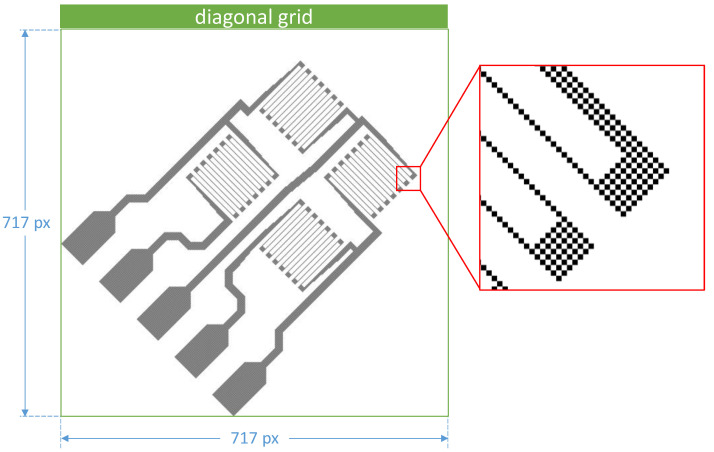
Print pattern according to Figure 2 rotated by 45° by using a diagonal grid.

For experimental validation, Figure 2 defines the underlying test structure used, which represents the aspects of perpendicularly oriented conductors in SG measurement grids in a realistic application-oriented design. The full-bridge SG from Figure 2 can be transferred to a diagonal grid according to the procedure in Figure 3 and, thus, be rotated. The result is shown in Figure 8. The grid is 717 rows and 717 columns, with 34,729 pixels filled in black. The number of black-filled pixels is identical to the full-bridge SG on the pixel grid (cf. Figure 2). The pattern shown in Figure 8 appears in a gray hue, although only black or white coloring is allowed for the pixels. The magnification section in Figure 8 shows that this is indeed the case and that a chessboard pattern has formed from previously black image areas due to the transformation. This pattern is indeed characteristic of raster graphics that have been transferred to a diagonal grid by rotation.

The white borders at the top and right edges of the image in Figure 8 do not contain any data relevant for printing and can optionally be removed. In fact, only a reduced image area of 660 columns and 659 lines is required for printing because it contains all data for material deposition. White borders may occur depending on the content of the source image if white areas are present in the image corners. For the full-bridge SG on a diagonal grid according to Figure 8, it can be stated that the required characteristics such as material quantity and material density distribution are preserved by the print pattern. To evaluate the electrical properties on diagonal grids of rotated full-bridge strain gages, a grid of full bridges without rotation at 500 DPI resolution (for reference) and full bridges with rotation at 707.107 DPI resolution are printed, respectively.

**Figure 9:**
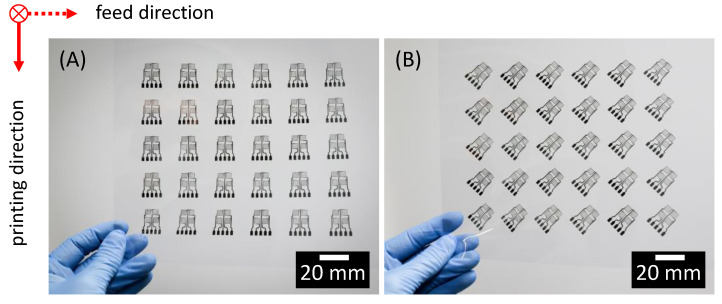
Photograph of printed and sintered SG full bridges on PEN film: (**A**) reference, not rotated, pixel grid; (**B**) rotated by 45°, diagonal grid.

The batch size is 30 each. For printing, the materials (silver ink on a PEN substrate) are used on the printer set-up (PiXDRO LP50) and sintered in a hot air oven for 30 min at 150 °C as described in chapter 2. Figure 9 shows a photo of the printed and sintered SG measurement grids without and with 45° rotation.

### 3.2. Optical Analysis

**Figure 10:**
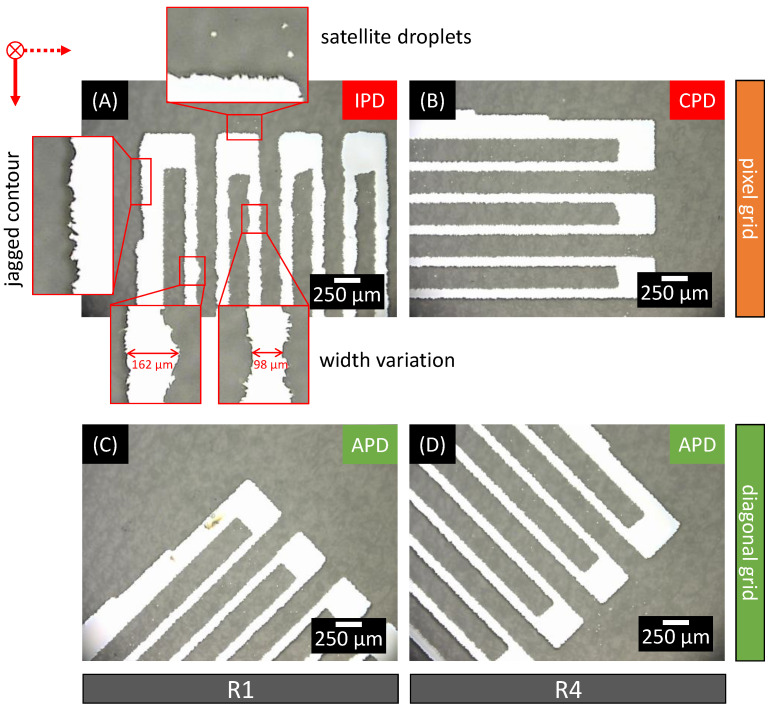
Microscopic images of SG full bridges on PEN substrate. Comparison of IPD (**A**) and CPD (**B**) printed conductors on a conventional pixel grid and APD; (**C**,**D**) printed conductors on a diagonal grid.

As a detailed view, microscopic image sections of the sintered measuring gratings are shown for each case in Figure 10. Print and feed directions are indicated by the red arrows, so that the influence on the material distribution can be observed depending on the measuring grid orientation.

Figure 10A shows a detailed section of a resistive element $R_{1}$ of the non-rotated measurement gratings. The printing direction coincides with the measuring conductor orientation, so that a measuring conductor is completely deposited during printing when the printhead passes over it (IPD). The time interval when adjacent material droplets impinge along the measuring conductor is thus considerably shorter than in the case of the measuring conductors in Figure 10B.

In Figure 10A, three main features can be observed.

1. The contour of the printed material, i.e., the transition of printed to blank surface, has a rough profile. The contour appears “jagged”. The reason for this is in the surface of the substrate, which has been pretreated by the manufacturer to improve the wetting properties for printing. Since this substrate-specific effect occurs equally for all prints and the hereby resulting lateral feature is significantly smaller than the width of the measuring conductors, a negligible influence on the electrical properties of the measuring conductor is assumed and the contour profile is not investigated further.
2. Very small material droplets are found between and around the printed structure in the printed image. The diameter of these droplets is even smaller than the feature shape of the contour profile. These satellite droplets are known to be a source of defects in inkjet printing and can lead to defects such as short circuits if they accumulate too much [8]. However, since the spacing of these droplets and their distance to the conductors are larger than their own diameter, the effect on the electrical properties of the sensing element is assumed to be negligible.
3. The measuring conductors and the feed conductor shown have an inhomogeneous width in the printed image. The width of the measuring conductors in the image section shown in Figure 10A varies by about 65% between 98 µm and 162 µm.

While the first two observations occur in all individual images in Figure 10, the print image of the measuring conductors differs. The minimum widths of the CPD printed measuring conductors (Figure 10B) are smaller than those of the IPD printed (Figure 10A) (87 µm in comparison to 98 µm) and show less variation in width (10% in comparison to 65%). This is an exemplary reproduction of the effect described in the state of the art and confirms that measuring conductors oriented perpendicular to the printing direction (CPD) have a smaller width [33].

The image sections of the 45° rotated measurement grid of $R_{1}$ and $R_{4}$ shown in Figure 10C, D differ in their orientation to the printing direction. The measurement gratings are not oriented in the printing direction and perpendicular to it but have an orientation of 45° to the printing direction (angled printing direction: APD). The time delay when adjacent material droplets impinge along the measuring guide is thus comparable to the measuring conductors in Figure 10B. This coincides with the optical impression of the measuring conductors with respect to their width. Both the width (values between 85 µm and 95 µm) and the variation of the width (about 11%) of the measuring conductors of the 45° rotated measuring grating correspond to those of the measuring conductors of perpendicular orientation (CPD) to the printing direction shown in Figure 10B.

The microscopy analysis is a first indication that a rotation by 45° leads to homogenization of the morphology of inkjet printed SG measurement gratings. The extent to which these results are also reflected in the electrical properties of the measuring gratings are investigated by further analysis.

### 3.3. Electrical Analysis

Determining the electrical properties of the printed and sintered measuring gratings provides important parameters for assessing the manufacturing quality. The electrical resistance of each individual quarter-bridge resistor element is measured using a digital multimeter.

Each sample comprises N=30 full bridges, each of which is generated during a single print job. Therefore, it is assumed that influencing factors of the environment (e.g., humidity, room temperature), the printing equipment (e.g., sedimentation of the ink or a changed droplet output) and the substrate (only one large substrate) exist as constant boundary conditions during a single print job. The two samples of non-rotated and rotated gratings are printed on two different days. Therefore, the influencing factors can change over days and absolute electrical measured values can only be compared within one print job. However, the relative variations of the electrical characteristics within a full bridge are significant for a comparison of the two different printing strategies since this is where the influence of the printing direction comes into play. The measure of bridge detuning of a Wheatstone bridge according to 2.5 can be used for this purpose. The N-fold realization of the test structure within a print job facilitates the statistical evaluation of the reproducibility and the accumulation of defective resistor elements.

**Figure 11:**
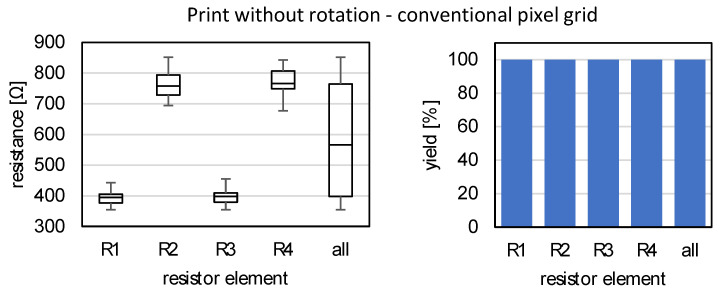
Electrical properties of SG full bridges, conventional print without rotation. **Left**: Resistances of the resistance elements, **right**: Yield of intact resistance elements.

Figure 11 (left) shows the absolute resistance values of the resistive elements for the measurement gratings without rotation. The measurements without rotation represent the benchmark for comparison to the 45° rotated full bridges on diagonal grids. By means of boxplots the statistical distribution of each of the 30 resistors $R_{1}$ to $R_{4}$ is given, as well as a boxplot for all 120 resistor elements together. In addition, the yield of intact resistor elements is shown in a second diagram (right). Not intact or defective elements are those for which no resistance can be determined with the multimeter in the measuring range of the measuring device. Typically, a faulty conductor is interrupted at one point by contamination or by a defect in the printed circuit. In the case of the SG measuring gratings described in Figure 11, all elements are intact, so the yield is 100%.

The evaluation broken down by resistors $R_{1}$ to $R_{4}$ shows a distinctive distribution. While the resistors $R_{1}$ and $R_{3}$ exhibit values of approx. 400 Ω, the values for the resistors $R_{2}$ and $R_{4}$ are approx. 800 Ω. Even considering the statistical variation, the value levels are clearly separated. These electrical properties are consistent with the effects observed in Figure 10A,B on the influence of the printing direction on the conductor orientation. The resistors $R_{1}$ and $R_{3}$ (cf. Figure 11) have in common that the printing direction corresponds to their measurement conductor orientation (IPD). They exhibit similar electrical properties. Similarly, resistors $R_{2}$ and $R_{4}$ can be assigned to a printing direction that is perpendicular to the measuring conductor orientation (CPD). The measured values obtained here indicate that the printing direction is an important factor influencing the electrical properties of inkjet-printed thin measuring conductors.

**Figure 12:**
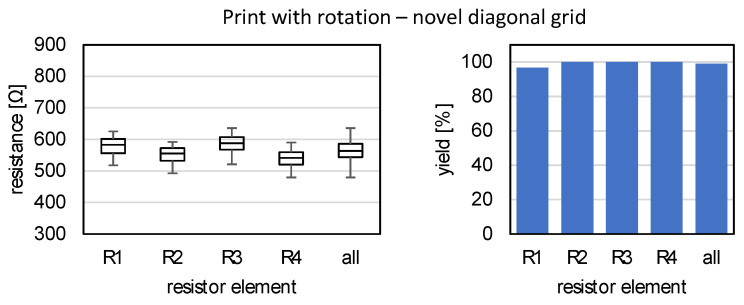
Electrical properties of SG full bridges, novel print with rotation using diagonal grids. **Left**: Resistances of the resistance elements; **Right**: Yield of intact resistance elements.

The electrical properties of the 45° rotated SG measuring gratings are to be compared. Figure 12 shows analogously the values of the yield (right) and the distribution of the resistance values (left) for the SG full bridges rotated by 45° by means of diagonal grids. Here, a defect occurred in one of the $R_{1}$ resistor elements, so that only N=119 are included in the evaluation of the resistors. Here, a different distribution of the values emerges. All resistances are in a range of approximately 500 Ω to 600 Ω. The difference between the measuring conductors $R_{1}$ and $R_{3}$ oriented in one direction and $R_{2}$ and $R_{4}$ perpendicular to it is still noticeable, but at a significantly reduced level. In addition, the resistances for $R_{1}$ and $R_{3}$ are higher than $R_{2}$ and $R_{4}$ in this case.

**Figure 13:**
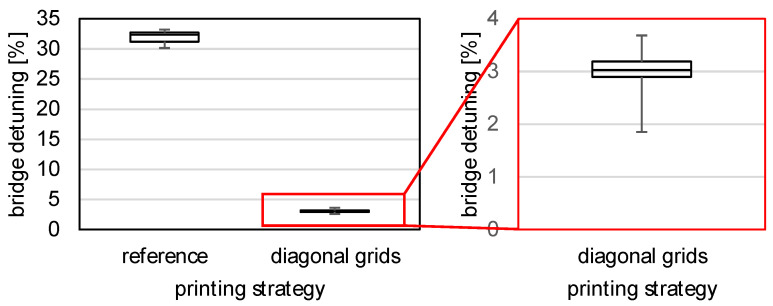
Detuning of SG full bridges.

To better place the measurement results in the application context of Wheatstone’s bridge circuit, the bridge detuning is determined for all full bridges to be fully evaluated and the result is shown as a boxplot in Figure 13 with N=30 samples for reference and N=29 for diagonal grids. While the bridge detuning without rotation is over 30%, the use of the diagonal grids for rotation by 45° was able to lower the level of detuning to about 3%.

### 3.4. Discussion

The use of a newly defined grid type for lossless image rotation for the preparation of printing patterns in inkjet printing fulfills the requirement for improving the manufacturing quality of inkjet-printed metallic measurement gratings in several respects: on the one hand, defined requirements for obtaining digital image information and, thus, the amount of material and material-density distribution to be deposited can be met. On the other hand, the experimental validation of the approach can show a measurable improvement of the electrical properties in terms of a reduction of the bridge detuning by one order of magnitude. This provides a solution that can be implemented purely digitally in additive manufacturing chains without having to address specific properties of the materials used, such as substrate or ink as coating material. Relevance is given, since many typical strain gauge configurations require measurement gratings with two mutually perpendicular measurement conductor orientations.

The effectiveness of the new proposed method has been shown, but the following practical issues should be considered when applying the method. The use of u–v grids is linked to a resolution adjustment factor of $\lambda \geq \sqrt{2}$ so that the set printing resolution must be increased significantly with increased rotation angles. The system technology of the inkjet printer used may only allow a limited increase in print resolution. In addition, many u–v grids require the setting of a non-integer resolution value, which must be supported by the printer hardware and software. Since the presented image rotation is linked to the arctangent function, an integer value for the rotation angle α is achieved in the case of a diagonal grid (u=v or α=45°). A list of rotation angles resulting from u–v grids with 1≤u,v≤20 as for the data points shown in Figure 7 is given in Table S1 in the Supplementary Material. Other typically used rotation angles of, e.g., 30° or 60° can be approximated.

A 11-19 grid leads to a rotation angle of α≈30.07° and a 19-11 grid to a rotation angle of α≈59.93°, both at a resolution adjustment factor of λ≈21.95. The symmetry visible in Figure 7 by swapping u and v becomes obvious in this example. Since a rotation grid for approximately 30° leads to a rotation grid for approximately 60° by swapping u and v for symmetry reasons, we will only talk about grids for 30° in the following examples. The angular deviation of ±0.07° using a 11-19 grid might be small enough to be acceptable for some use cases, but the corresponding resolution adjustment factor of λ≈21.95 might be too much. While using a 7-12 grid reduces the resolution adjustment factor to λ≈13.89, the angular deviation increases to ±0.26°. Furthermore a 3-5 grid reduces the resolution adjustment factor to λ≈5.83 at an angular deviation of ±0.96°. Depending on the use case, tolerances in the rotation angle and an acceptable resolution adjustment factor, different rotation grids might be feasible.

While suitable applications must be found for many types of u–v grids, the obvious benefit for a 1-1 grid (45°) has already been explored in this study for SG full bridges. Besides a reduction of the bridge detuning, the resistances of all conductor elements vary less compared to the reference sample at 0°. However, the resistive elements $R_{1}$ and $R_{3}$ are at a slightly higher ohmic level compared to $R_{2}$ and $R_{4}$. Possible causes may be found in the design of the print pattern which is systematic for all measured values. They are determined using probe tips on the printed contact pads. All printed different conductor types like the contact pads, the feed, turnaround and measuring conductors sum up their different fractions to the total resistivity. The formation of the printed metallic film may influence the measured values according to the design of the print pattern. Further investigations on the overall film formation may be beneficial for an improved understanding of such print patterns and the influence on the electrical properties.

## 4. Conclusions

In summary, lossless image rotation on print patterns as used for SGs can affect their electrical properties. One possible reason for this is the resulting change in the time interval in which adjacent material droplets impinge along narrow conductive paths. The lowering of the bridge detuning of inkjet-printed full-bridge SG measuring gratings of two different measuring conductor orientations by one order of magnitude can be an important step towards significantly improving the manufacturing quality and thus bringing digitally and additively manufactured SGs further into use. Values for the detuning of conventionally manufactured SG full bridges are a few to less than one percent. While bridge detuning of more than 30% makes the use of printed sensing gratings unattractive for many applications, sensing gratings of a few percent detuning can already be used in a variety of ways and provide a good basis to further improve the electrical properties using methods such as laser trimming by cutting off material and, thus, adjusting the conductor cross-section to bring them to the same order of magnitude as conventionally fabricated sensors.

For further validation, an analysis and evaluation of the sensory and mechano-resistive properties is desirable. Since the methods used in this work are designed to analyze the individual resistive elements and their effect on bridge detuning, the design of the printing pattern and the substrate material are chosen accordingly to reduce interfering boundary conditions. To determine the performance under strain and the influence of the methods presented here, further investigations using adapted printing patterns are required to reduce disturbing influences (e.g., additional external wiring). Such grating patterns that interconnect the resistive elements in a circle at the same time do not allow analysis of the individual resistive elements. In addition, the choice of materials used must be coordinated with the additional layers required, such as adhesive and protective layers for covering, because they can significantly influence the measurement results. Since the systematics and experimental design required for this differ significantly from the methodology of this work and a variety of characterizations should be performed, this is not covered within the scope of this work.

There is a need for further investigations to gain a fundamental understanding of the influence of this approach on layer formation. The choice of substrate material, as well as the size and complexity of the printed structure, does not allow for a robust analysis of the layer morphology within the scope of the work performed. Deformations of the substrate due to thermal influence cannot be fully compensated due to the size of the test structure and remaining systematic errors may lead to inaccurate interpretations. Therefore, further investigations with an adapted test design and reduced printing patterns are required to be able to evaluate the layer formation along the narrow measurement conductors in detail.

By applying the new method presented and validated here, a significant increase in the manufacturing quality of SGs is expected for inkjet printing technology. Validation for different materials and combinations of inks and substrates needs to be performed. Further understanding of the mechanisms of how layer formation of complex structures such as SGs occurs also needs to be established. This will require volumetric depth information in addition to data from profile measurements of the surface of printed particle layers. These volumetric data can contribute to a model to simulate the achievable electrical properties, so that new insights into the influence and mode of action of material deposition, but also sintering on different regimes of time and temperature gradients may be found.

## Figures and Tables

**Table 1:** Values u, v, rotation angle α and resolution adjustment factor λ for examples (A) to (F) according to Figure 4.

Example *	u [-]	v [-]	*α* [°]	*λ* [-]
(A)	1	1	45	$\sqrt{2}\;∼\;1.414$
(B)	1	2	∼26.57	$\sqrt{5}\;∼\;2.236$
(C)	2	1	∼63.43	$\sqrt{5}\;∼\;2.236$
(D)	2	2	45	$\sqrt{8}\;∼\;2.828$
(E)	1	3	∼18.43	$\sqrt{10}\;∼\;3.162$
(F)	3	4	∼36.87	$\sqrt{25}=5$

* Example identifier according to Figure 4 and Figure 5.

## Data Availability

The data presented in this study are available on request from the corresponding author.

## Supplementary Materials

The following supporting information can be downloaded at: https://www.mdpi.com/article/10.3390/s22166119/s1, Table S1: Rotation angle α and resolution adjustment factor λ for values u, v≤20.
