# Supplementary material for: Rotation Grids for Improved Electrical Properties of Inkjet-Printed Strain Gauges
Source: Sensors (Basel). 2022 Aug 16;22(16):6119. doi: 10.3390/s22166119 (PMC9415692; doi:10.3390/s22166119)
Supplement: Supplementary file 1 [file sensors-22-06119-s001.zip › sensors-1847054-supplementary.pdf]

Supporting Information

Rotation grids for improved electrical properties of inkjet-printed strain gauges

Matthias Rehberger [1,*], Jonas Mertin [1], Christian Vedder [1], Jochen Stollenwerk [1] and Johannes Henrich Schleifenbaum [2]

[1] Fraunhofer Institute for Laser Technology ILT, 52074 Aachen, Germany
[2] Digital Additive Production (DAP), RWTH Aachen University, 52074 Aachen, Germany;
* Correspondence: matthias_rehberger@web.de;

Table S1. Values u. v. rotation angle α and resolution adjustment factor λ for values u. $v \leq 20$.

u	v	λ	α
1	1	1.41	45.00
1	2	2.24	26.57
1	3	3.16	18.43
1	4	4.12	14.04
1	5	5.10	11.31
1	6	6.08	9.46
1	7	7.07	8.13
1	8	8.06	7.13
1	9	9.06	6.34
1	10	10.05	5.71
1	11	11.05	5.19
1	12	12.04	4.76
1	13	13.04	4.40
1	14	14.04	4.09
1	15	15.03	3.81
1	16	16.03	3.58
1	17	17.03	3.37
1	18	18.03	3.18
1	19	19.03	3.01
1	20	20.02	2.86
2	1	2.24	63.43
2	2	2.83	45.00
2	3	3.61	33.69
2	4	4.47	26.57
2	5	5.39	21.80
2	6	6.32	18.43
2	7	7.28	15.95
2	8	8.25	14.04
2	9	9.22	12.53
2	10	10.20	11.31
2	11	11.18	10.30
2	12	12.17	9.46
2	13	13.15	8.75
2	14	14.14	8.13
2	15	15.13	7.59
2	16	16.12	7.13
2	17	17.12	6.71
2	18	18.11	6.34
2	19	19.10	6.01
2	20	20.10	5.71
3	1	3.16	71.57
3	2	3.61	56.31
3	3	4.24	45.00
3	4	5.00	36.87
3	5	5.83	30.96
3	6	6.71	26.57
3	7	7.62	23.20
3	8	8.54	20.56
3	9	9.49	18.43
3	10	10.44	16.70

u	v	λ	α
3	11	11.40	15.26
3	12	12.37	14.04
3	13	13.34	12.99
3	14	14.32	12.09
3	15	15.30	11.31
3	16	16.28	10.62
3	17	17.26	10.01
3	18	18.25	9.46
3	19	19.24	8.97
3	20	20.22	8.53
4	1	4.12	75.96
4	2	4.47	63.43
4	3	5.00	53.13
4	4	5.66	45.00
4	5	6.40	38.66
4	6	7.21	33.69
4	7	8.06	29.74
4	8	8.94	26.57
4	9	9.85	23.96
4	10	10.77	21.80
4	11	11.70	19.98
4	12	12.65	18.43
4	13	13.60	17.10
4	14	14.56	15.95
4	15	15.52	14.93
4	16	16.49	14.04
4	17	17.46	13.24
4	18	18.44	12.53
4	19	19.42	11.89
4	20	20.40	11.31
5	1	5.10	78.69
5	2	5.39	68.20
5	3	5.83	59.04
5	4	6.40	51.34
5	5	7.07	45.00
5	6	7.81	39.81
5	7	8.60	35.54
5	8	9.43	32.01
5	9	10.30	29.05
5	10	11.18	26.57
5	11	12.08	24.44
5	12	13.00	22.62
5	13	13.93	21.04
5	14	14.87	19.65
5	15	15.81	18.43
5	16	16.76	17.35
5	17	17.72	16.39
5	18	18.68	15.52
5	19	19.65	14.74
5	20	20.62	14.04

u	v	λ	α
6	1	6.08	80.54
6	2	6.32	71.57
6	3	6.71	63.43
6	4	7.21	56.31
6	5	7.81	50.19
6	6	8.49	45.00
6	7	9.22	40.60
6	8	10.00	36.87
6	9	10.82	33.69
6	10	11.66	30.96
6	11	12.53	28.61
6	12	13.42	26.57
6	13	14.32	24.78
6	14	15.23	23.20
6	15	16.16	21.80
6	16	17.09	20.56
6	17	18.03	19.44
6	18	18.97	18.43
6	19	19.92	17.53
6	20	20.88	16.70
7	1	7.07	81.87
7	2	7.28	74.05
7	3	7.62	66.80
7	4	8.06	60.26
7	5	8.60	54.46
7	6	9.22	49.40
7	7	9.90	45.00
7	8	10.63	41.19
7	9	11.40	37.87
7	10	12.21	34.99
7	11	13.04	32.47
7	12	13.89	30.26
7	13	14.76	28.30
7	14	15.65	26.57
7	15	16.55	25.02
7	16	17.46	23.63
7	17	18.38	22.38
7	18	19.31	21.25
7	19	20.25	20.22
7	20	21.19	19.29
8	1	8.06	82.87
8	2	8.25	75.96
8	3	8.54	69.44
8	4	8.94	63.43
8	5	9.43	57.99
8	6	10.00	53.13
8	7	10.63	48.81
8	8	11.31	45.00
8	9	12.04	41.63
8	10	12.81	38.66

u	v	λ	α
8	11	13.60	36.03
8	12	14.42	33.69
8	13	15.26	31.61
8	14	16.12	29.74
8	15	17.00	28.07
8	16	17.89	26.57
8	17	18.79	25.20
8	18	19.70	23.96
8	19	20.62	22.83
8	20	21.54	21.80
9	1	9.06	83.66
9	2	9.22	77.47
9	3	9.49	71.57
9	4	9.85	66.04
9	5	10.30	60.95
9	6	10.82	56.31
9	7	11.40	52.13
9	8	12.04	48.37
9	9	12.73	45.00
9	10	13.45	41.99
9	11	14.21	39.29
9	12	15.00	36.87
9	13	15.81	34.70
9	14	16.64	32.74
9	15	17.49	30.96
9	16	18.36	29.36
9	17	19.24	27.90
9	18	20.12	26.57
9	19	21.02	25.35
9	20	21.93	24.23
10	1	10.05	84.29
10	2	10.20	78.69
10	3	10.44	73.30
10	4	10.77	68.20
10	5	11.18	63.43
10	6	11.66	59.04
10	7	12.21	55.01
10	8	12.81	51.34
10	9	13.45	48.01
10	10	14.14	45.00
10	11	14.87	42.27
10	12	15.62	39.81
10	13	16.40	37.57
10	14	17.20	35.54
10	15	18.03	33.69
10	16	18.87	32.01
10	17	19.72	30.47
10	18	20.59	29.05
10	19	21.47	27.76
10	20	22.36	26.57

u	v	λ	α
11	1	11.05	84.81
11	2	11.18	79.70
11	3	11.40	74.74
11	4	11.70	70.02
11	5	12.08	65.56
11	6	12.53	61.39
11	7	13.04	57.53
11	8	13.60	53.97
11	9	14.21	50.71
11	10	14.87	47.73
11	11	15.56	45.00
11	12	16.28	42.51
11	13	17.03	40.24
11	14	17.80	38.16
11	15	18.60	36.25
11	16	19.42	34.51
11	17	20.25	32.91
11	18	21.10	31.43
11	19	21.95	30.07
11	20	22.83	28.81
12	1	12.04	85.24
12	2	12.17	80.54
12	3	12.37	75.96
12	4	12.65	71.57
12	5	13.00	67.38
12	6	13.42	63.43
12	7	13.89	59.74
12	8	14.42	56.31
12	9	15.00	53.13
12	10	15.62	50.19
12	11	16.28	47.49
12	12	16.97	45.00
12	13	17.69	42.71
12	14	18.44	40.60
12	15	19.21	38.66
12	16	20.00	36.87
12	17	20.81	35.22
12	18	21.63	33.69
12	19	22.47	32.28
12	20	23.32	30.96
13	1	13.04	85.60
13	2	13.15	81.25
13	3	13.34	77.01
13	4	13.60	72.90
13	5	13.93	68.96
13	6	14.32	65.22
13	7	14.76	61.70
13	8	15.26	58.39
13	9	15.81	55.30
13	10	16.40	52.43

u	v	λ	α
13	11	17.03	49.76
13	12	17.69	47.29
13	13	18.38	45.00
13	14	19.10	42.88
13	15	19.85	40.91
13	16	20.62	39.09
13	17	21.40	37.41
13	18	22.20	35.84
13	19	23.02	34.38
13	20	23.85	33.02
14	1	14.04	85.91
14	2	14.14	81.87
14	3	14.32	77.91
14	4	14.56	74.05
14	5	14.87	70.35
14	6	15.23	66.80
14	7	15.65	63.43
14	8	16.12	60.26
14	9	16.64	57.26
14	10	17.20	54.46
14	11	17.80	51.84
14	12	18.44	49.40
14	13	19.10	47.12
14	14	19.80	45.00
14	15	20.52	43.03
14	16	21.26	41.19
14	17	22.02	39.47
14	18	22.80	37.87
14	19	23.60	36.38
14	20	24.41	34.99
15	1	15.03	86.19
15	2	15.13	82.41
15	3	15.30	78.69
15	4	15.52	75.07
15	5	15.81	71.57
15	6	16.16	68.20
15	7	16.55	64.98
15	8	17.00	61.93
15	9	17.49	59.04
15	10	18.03	56.31
15	11	18.60	53.75
15	12	19.21	51.34
15	13	19.85	49.09
15	14	20.52	46.97
15	15	21.21	45.00
15	16	21.93	43.15
15	17	22.67	41.42
15	18	23.43	39.81
15	19	24.21	38.29
15	20	25.00	36.87

u	v	λ	α
16	1	16.03	86.42
16	2	16.12	82.87
16	3	16.28	79.38
16	4	16.49	75.96
16	5	16.76	72.65
16	6	17.09	69.44
16	7	17.46	66.37
16	8	17.89	63.43
16	9	18.36	60.64
16	10	18.87	57.99
16	11	19.42	55.49
16	12	20.00	53.13
16	13	20.62	50.91
16	14	21.26	48.81
16	15	21.93	46.85
16	16	22.63	45.00
16	17	23.35	43.26
16	18	24.08	41.63
16	19	24.84	40.10
16	20	25.61	38.66
17	1	17.03	86.63
17	2	17.12	83.29
17	3	17.26	79.99
17	4	17.46	76.76
17	5	17.72	73.61
17	6	18.03	70.56
17	7	18.38	67.62
17	8	18.79	64.80
17	9	19.24	62.10
17	10	19.72	59.53
17	11	20.25	57.09
17	12	20.81	54.78
17	13	21.40	52.59
17	14	22.02	50.53
17	15	22.67	48.58
17	16	23.35	46.74
17	17	24.04	45.00
17	18	24.76	43.36
17	19	25.50	41.82
17	20	26.25	40.36
18	1	18.03	86.82
18	2	18.11	83.66
18	3	18.25	80.54
18	4	18.44	77.47
18	5	18.68	74.48
18	6	18.97	71.57
18	7	19.31	68.75
18	8	19.70	66.04
18	9	20.12	63.43
18	10	20.59	60.95

u	v	λ	α
18	11	21.10	58.57
18	12	21.63	56.31
18	13	22.20	54.16
18	14	22.80	52.13
18	15	23.43	50.19
18	16	24.08	48.37
18	17	24.76	46.64
18	18	25.46	45.00
18	19	26.17	43.45
18	20	26.91	41.99
19	1	19.03	86.99
19	2	19.10	83.99
19	3	19.24	81.03
19	4	19.42	78.11
19	5	19.65	75.26
19	6	19.92	72.47
19	7	20.25	69.78
19	8	20.62	67.17
19	9	21.02	64.65
19	10	21.47	62.24
19	11	21.95	59.93
19	12	22.47	57.72
19	13	23.02	55.62
19	14	23.60	53.62
19	15	24.21	51.71
19	16	24.84	49.90
19	17	25.50	48.18
19	18	26.17	46.55
19	19	26.87	45.00
19	20	27.59	43.53
20	1	20.02	87.14
20	2	20.10	84.29
20	3	20.22	81.47
20	4	20.40	78.69
20	5	20.62	75.96
20	6	20.88	73.30
20	7	21.19	70.71
20	8	21.54	68.20
20	9	21.93	65.77
20	10	22.36	63.43
20	11	22.83	61.19
20	12	23.32	59.04
20	13	23.85	56.98
20	14	24.41	55.01
20	15	25.00	53.13
20	16	25.61	51.34
20	17	26.25	49.64
20	18	26.91	48.01
20	19	27.59	46.47
20	20	28.28	45.00
